# Structural Insights into Separase Architecture and Substrate Recognition through Computational Modelling of Caspase-Like and Death Domains

**DOI:** 10.1371/journal.pcbi.1004548

**Published:** 2015-10-29

**Authors:** Anja Winter, Ralf Schmid, Richard Bayliss

**Affiliations:** Department of Biochemistry, University of Leicester, Leicester, United Kingdom; Wake Forest University, UNITED STATES

## Abstract

Separases are large proteins that mediate sister chromatid disjunction in all eukaryotes. They belong to clan CD of cysteine peptidases and contain a well-conserved C-terminal catalytic protease domain similar to caspases and gingipains. However, unlike other well-characterized groups of clan CD peptidases, there are no high-resolution structures of separases and the details of their regulation and substrate recognition are poorly understood. Here we undertook an in-depth bioinformatical analysis of separases from different species with respect to their similarity in amino acid sequence and protein fold in comparison to caspases, MALT-1 proteins (*m*ucosa-*a*ssociated *l*ymphoid*t*issue lymphoma translocation protein *1*) and gingipain-R. A comparative model of the single C-terminal caspase-like domain in separase from *C*. *elegans* suggests similar binding modes of substrate peptides between these protein subfamilies, and enables differences in substrate specificity of separase proteins to be rationalised. We also modelled a newly identified putative death domain, located N-terminal to the caspase-like domain. The surface features of this domain identify potential sites of protein-protein interactions. Notably, we identified a novel conserved region with the consensus sequence WWxxRxxLD predicted to be exposed on the surface of the death domain, which we termed the WR motif. We envisage that findings from our study will guide structural and functional studies of this important protein family.

## Author Summary

The separation of sister chromatids is a crucial step in cell division and is triggered by the activation of separase, a protease that cleaves the proteins that maintain the cohesion between sister chromatids. Knowledge of the molecular structure and activation mechanism of separase is limited by the difficulty of obtaining structural information on this large and flexible protein. Sequence conservation between separase homologues from diverse species is limited to the C-terminal region that contains the catalytically active protease domain. We conducted an in-depth bioinformatical analysis of separase and generated structural models of the two conserved domains that comprise the C-terminal region: a caspase-like domain and a putative death domain. This analysis provided insights into substrate recognition and identified potential sites of protein-protein interactions. Both the death domain and caspase-like domain are well-conserved in separases, which suggests an evolutionary pressure to keep these two domains together, perhaps to enable separase activity and/or provide stability. Insights into the molecular structures of separase gained in this study may provide a starting point for experimental structural studies on this protein and may aid therapeutic development against cancers where chromosomes are improperly segregated.

## Introduction

Separase overexpression and aberrant nuclear localization are reported in a broad range of human tumours, and its overexpression in mouse models results in tumourigenesis [1, 2]. A strong correlation has been made between overexpression of separase protein in adult glioblastoma and a high incidence of relapse and reduced overall survival [3]. Furthermore, abnormal separase expression and mislocalisation are drivers of aneuploidy and tumourigenesis [4]. Separase has a crucial role during mitosis, namely the mediation of sister chromatid disjunction at the onset of anaphase by cleavage of one of the subunits of the cohesin complex, Scc1 [5–8]. The cleavage of Scc1 by separase requires DNA or RNA, suggesting that the DNA binding activity of separase may be important for its ability to cleave cohesin [9]. Separase is also involved in centriole disengagement by cleavage of kendrin, also named pericentrin, at a separase consensus site (SxExxR) [10–13]. Its prevalence in a number of cancers led to its recognition as prime candidate to target chromosomal missegregation-induced tumorigenesis in cancer therapies [3, 14]. Recently, a noncompetitive inhibitor of separase, Sepin-1, was characterized, which can inhibit the growth of cancer cell lines and mammary xenograft tumors in mice by inducing apoptosis [14].

Throughout the cell cycle, separase forms a complex with its inhibitor securin which binds to the N-terminal part of separases while preventing access to the catalytic site [15–17]. This interaction is resolved in anaphase when securin is degraded by the anaphase-promoting complex (APC) [18]. The catalytic activity of separases resides in their well-conserved C-terminal part, a region predicted to contain a protease domain common to caspases [8]. Separases are large proteins with molecular weights ranging from 140–240 kDa, apart from a few exceptions (*Encephalitozoon* and *Drosophila* species). However, in *Drosophila*, separase is made up of two genes, *sse* which is homologous to the C-terminal part of separases from other species and *thr*, which is homologous to the N-terminal part [19]. The products of these two genes have a combined molecular weight of ~220 kDa. It remains to be seen whether *Encephalitozoon* species with apparently smaller separase proteins also encode for another protein homologous to the N-terminal region of separases.

Separases belong to clan CD of cysteine peptidases and are related to the clostripain, metacaspase, paracaspase, caspase and gingipain families [20–23]. Each family shares ~25% sequence identity with all other families and the highest level of sequence identity is found around the active site residues. CD clan peptidases possess strictly conserved, catalytic histidine and cysteine residues in their C-terminal domain [8, 24]. In caspases, MALT-1 (*m*ucosa-*a*ssociated *l*ymphoid*t*issue lymphoma translocation protein *1*) and gingipain, this catalytic dyad is brought into juxtaposition by association of two hydrophobic β-strands [25–28]. Viadiu and co-workers reported that, according to their bioinformatical analysis of human separase, the protein contains not only one but two C-terminal caspase-like domains, of which only the more C-terminal one is active [29]. In addition to the caspase-like C-terminal domain, separases also contain a non-conserved N-terminal domain, which is thought to consist of Armadillo (ARM) or HEAT repeat motifs [19, 29]. The C-terminal domain is separated from the N-terminal region by an unstructured central stretch (a 'hinge region') [29]. Pull-down studies revealed that the N and C- terminal regions of both human and budding yeast separase interact with each other [17, 19]. Moreover, in yeast the entire N-terminal region seems to be required for catalytic activity of the C-terminal caspase-like domain [17].

Caspases recognize the acidic residue aspartate in P1 position, whereas gingipain-R and the paracaspase MALT-1 are specific for arginine in that position. Separases cleave their substrate Scc1 at two related sites, both with an arginine in the P1 position [7, 30, 31]. Mutating either of these residues abolishes cleavage at that site but is not lethal to cells. However mutating both sites is lethal and prevents sister chromatid segregation [7]. Separases also possess autocleavage sites which produce a shorter version that retains activity, as has been shown for separase from *X*. *laevis* and humans [32–35]. Cleavage site analysis has revealed that separases from budding and fission yeast cleave the core motif (D/E)xxR [30, 36], a consensus sequence similar to those recognized by separases from mammals, birds and reptiles, ExxR [33].

Despite a plethora of information about separases’ involvement in the cell cycle, little is known about their structure and function at a molecular level. This is mainly due to an inherent instability of separases without their inhibitor securin, and the flexibility of the separase-securin complex. Most publications to date investigate separase in a cellular context with its inhibitor securin present. In order to gain insights into the structural make-up of separases, their domain organisation and their interaction with securin, we undertook an in-depth bioinformatical analysis of separases. We provide evidence for novel, highly conserved regions in separase that may be important for the overall three-dimensional arrangement of the domains. We also provide a homology model of a separase caspase-like catalytic domain and suggest how a substrate peptide might bind in its active site.

## Results

### Secondary structure prediction and multiple sequence alignment

We first examined separases from *H*. *sapiens*, *A*. *thaliana*, *S*. *cerevisiae* and *C*. *elegans* using GLOBPLOT2 [37]. GLOBPLOT2 predicts disordered regions based on propensities for amino acids to be either in regular secondary structures (α-helices or β-strands) or outside of them (Russell/Linding propensities). The separase proteins examined generally appear to be ordered, globular proteins with only a few disordered regions. For the separase from *C*. *elegans*, one disordered stretch around residue 400 and another at the far C-terminus in the catalytic domain were predicted. Similar results were obtained from *S*. *cerevisiae* (disordered region around residue 950), *A*. *thaliana* (disordered regions around residues 410 and 1110) and *H*. *sapiens* (disordered regions around residues 1300–1350, 1450–1600 (natural cleavage site), 2020 (around catalytic site). This indicates that separases are globular proteins with a possible disordered region in the N-terminal region. This region might divide the N-terminal portion from the remainder of the protein and act as a ‘hinge region’, as has been observed in electron micrographs of the human separase-secuin complex [29].

**Fig 1:**
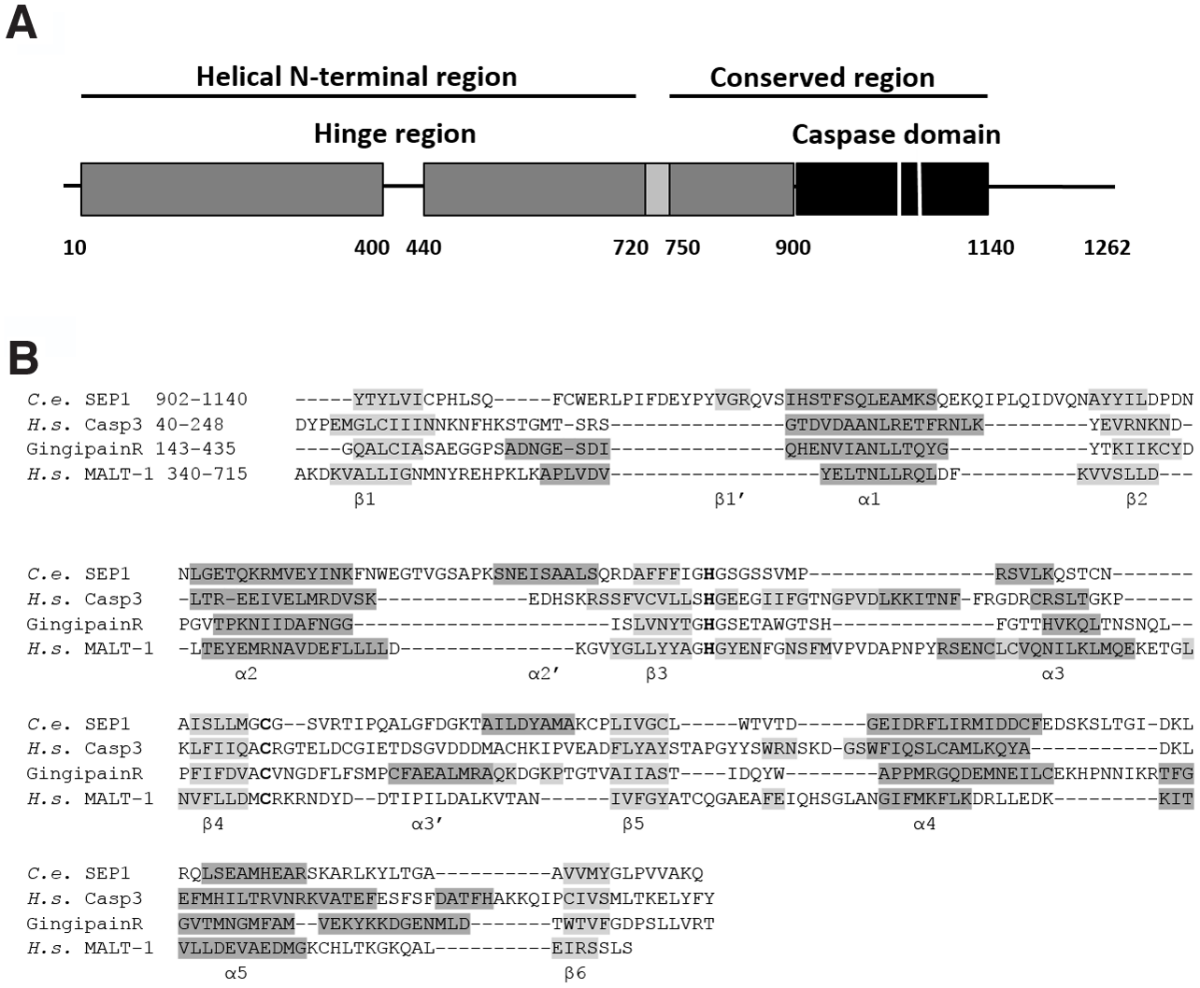
Separases from all species share a similar topology with highly conserved regions, which includes a caspase-like domain in their C-terminal region. (A) Consolidated secondary structure prediction of separase from *C*. *elegans* using both PsiPred and JPred predicts a largely helical N-terminus (dark grey) with an unstructured region around residue 400 and a region of three β-strands from residues 720 to 750 (light grey). The conserved C-terminal half harbours the caspase-like domain (black), residues 900 to 1140. The catalytic dyad is indicated as white lines. (B) Multiple sequence alignment of the caspase-like domain of separase from *C*. *elegans* in comparison with human caspase 3, gingipain-R and MALT-1. Alignment was manually adjusted using Jalview to match secondary structure elements from predictions (PsiPred) of separases to structural elements as observed in caspase 3 (3EDQ), MALT-1 (3UO8) and gingipain R (1CVR). α-helices are shown in dark grey and β-strands in light grey. The conserved catalytic dyad (C, H) is shown in bold letters.

Next we set out to survey the sequence conservation among separase homologues from a wide range of taxa and predict the domain structure of the proteins using a comprehensive bioinformatical analysis. By combining multiple sequence alignments with secondary structure predictions we were able to identify several regions of separase that are well conserved (Fig 1A). Care was taken to avoid biasing the alignment towards a single taxonomic branch by aligning a representative set of separase protein sequences. The alignment revealed little sequence conservation in the N-terminal region of all proteins analysed. In all sequences, this region is predicted to consist of α-helices that do not appear to be conserved in either length or relative position within the respective protein sequence. This region is generally followed by a disordered region (residues 400 to 440 in *C*. *elegans* separase) as well as three β-strands (residues 720 to 750 in *C*. *elegans* separase). In some homologues these predicted β-strands were absent, only two strands predicted or interspersed with short helices. Finally, a well-conserved C-terminal region of approximately 240 amino acids was identified in all homologues that also contains the catalytically active residues histidine and cysteine (Fig 1A for a topological overview and S1 File).

**Fig 2:**
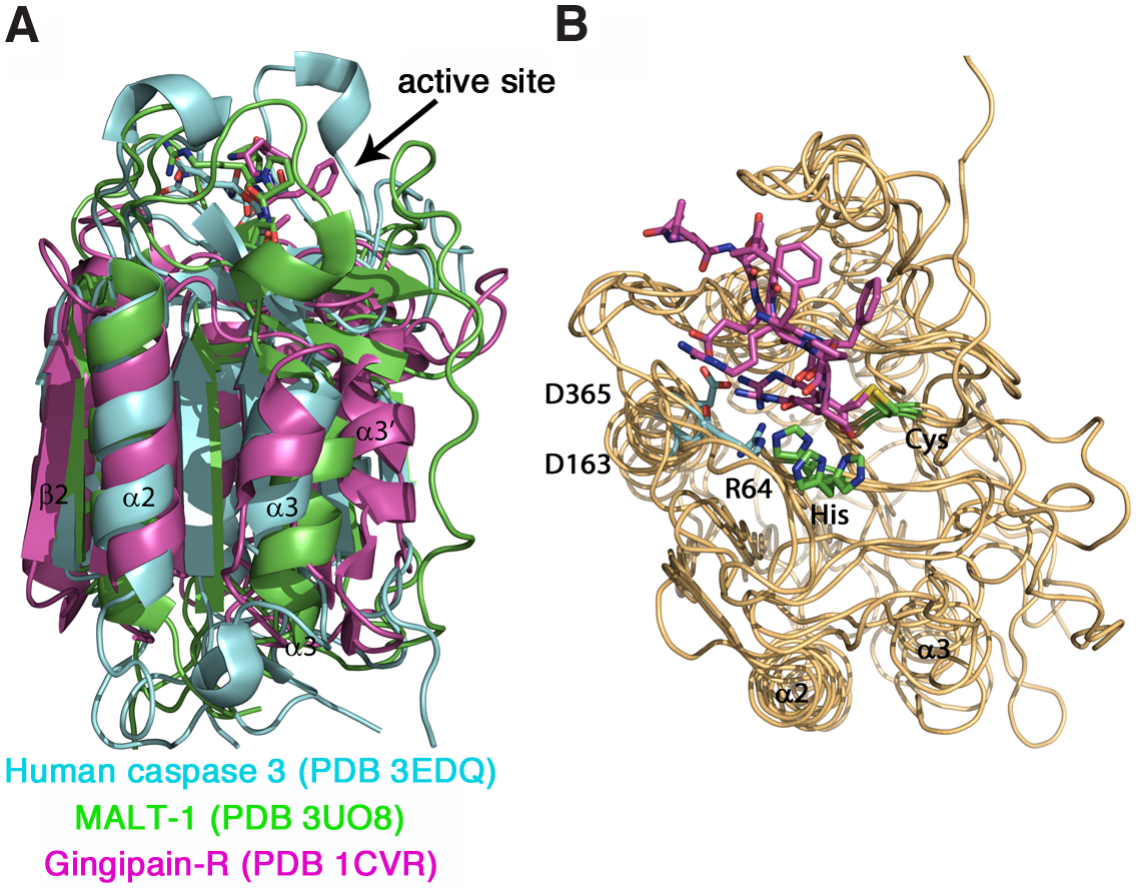
Comparison of the structures of human caspase 3, human MALT-1 and gingipain-R. (A) Overlaid structures of human caspase 3 (cyan, PDB code 3EDQ), human MALT-1 (green, PDB code 3UO8) and gingipain-R (magenta, PDB code 1CVR) show a similar overall fold consisting of a central six-stranded β -sheet flanked by α-helices. In gingipain-R, a helix connects the first four with the last two β-strands and replaces the inter-subunit linker present in caspases. In caspases the first four β -strands belong to the larger subunit p20 and the remaining two β-strands to the smaller subunit p10. The C-terminal Ig domain of MALT-1, subdomain A and the C-terminal IgSF domain of gingipain-R were omitted for clarity. (B) Comparison of the substrate binding pockets reveal a very similar binding mode of peptide inhibitors ace-LDESD-cho (for caspase 3), z-VRPR-fmk (MALT-1) and FFR-cmk (gingipain-R). Peptide inhibitors (magenta) and catalytic residues His and Cys (green) are shown as sticks. Residues mainly responsible for the recognition of the respective peptide P1 are shown as cyan sticks: Arg64 (for caspase 3), Asp163 (for gingipain-R) and Asp365 (for MALT-1). Figures were prepared with PyMol.

It is widely appreciated that protein three-dimensional structure is more conserved than either sequence or function within protein families [38]. Hence we included the closely related proteins gingipain-R and caspases in the alignment to ensure that the structural similarities between these related proteins are reflected in the alignment (Fig 1B). We first aligned the sequences of members of different CD clan protease families such as gingipain-R, human caspase 1, 3 and 7 and caspase 1 from *Spodoptera frugiperda* to reveal sequence conservation and similarities in secondary structure elements. The catalytic residues of each homologue were aligned, and the alignment then built up by matching stretches with hydrophobic or hydrophilic amino acids. The alignment was adjusted by aligning secondary structure elements from available crystal structures. Superimposing crystal structures from gingipain R (PDB code 1CVR) and human caspase 3 (PDB code 3EDQ) reveals that both structures are very similar (Fig 2A). Caspases generally crystallise as a dimer of a heterodimer, which consists of the smaller (p10) and larger (p20) caspase subunit. Both subunits are mainly held together by the interdomain linker. In our view, the heterodimer of caspases represents a folding unit, and we treated this dimer as one consecutive amino acid sequence in our alignment. This notion is further supported by the striking similarity in overall fold of this heterodimer to the caspase-like domain of gingipain R (subdomain B, amino acids 144 to 341) and caspase-like domain of MALT-1. Our alignment was further populated with representative separase sequences of different taxonomic branches. Overall, the alignment reveals a high degree of conservation both at amino acid sequence level as well as within the order of predicted secondary structure elements (see S1 File for a full alignment). This led us to conclude that separases have one conserved C-terminal region that is very similar to the joint smaller and larger subunits of caspases (p10 and p20, respectively) and gingipain R, subdomain B [25] and thus possesses a single caspase-like fold.

### The C-terminal catalytic region of separases is structurally similar to the caspase heterodimer and subdomain B of gingipain R

To gain information about the likely organisation of secondary structure elements in separase we compared secondary structure predictions of separase with structural information from caspases. Structurally, a caspase heterodimer is formed of a small and a large subunit that together form a central β-sheet comprising six β-strands [39]. This β-sheet is flanked by α-helices. The sequence of structural elements is: β1, α1, β2, α2, β3, α3, β4, β5, α4, α5, β6 where β1, α1, β2, α2, β3, α3 and β4 are part of the p20 subunit whereas β5, α4, α5, β6 are part of the p10 subunit (Fig 1B). Caspase 3 also contains three short β-strands between amino acids 122 and 135 (denominated β_I_, β_II_, β_III_ in [39]), which are replaced by a long loop in the gingipain-R structure. The catalytic residues are located just C-terminal of β-strands 3 (His) and 4 (Cys), respectively. Strikingly this sequence of secondary structure elements is also predicted in the C-terminal region of all separase homologues that we analysed. The only additions in separases are β1’ which is located between β1 and α1, helix α2’, which is located between α2 and β3 and helix α3’, which is located between β4 and β5 (Fig 1B). Notably, the additional helix α3’ is also present in gingipain R and connects the first four parallel β-strands to the following two anti-parallel β-strands β9 and β10. In caspases this region is called the intersubunit linker where the two subunits interact mainly via hydrogen bonding [39]. Consolidated secondary structure predictions of the C-terminal region of separases using both PsiPred [40] and JPred [41] revealed high similarities in the sequence and position of secondary structure elements among separase homologues as well as between caspases and gingipain R. Integrating multiple sequence alignment, secondary structure prediction and structural information highlights not only the region around the catalytic dyad but also the six buried β-strands as well-conserved. This led us to the following conclusions: i. there is only one caspase-like domain in separases, which corresponds to the functional heterodimer (p20 plus p10 subunit) of caspases; ii. this domain consists of six β-strands that are flanked by five α-helices (Fig 1B); iii. an additional helix in separases, helix α3’, connects the first four β-strands to the last two instead of the intersubunit linker in caspases.

### Comparative modelling of the C-terminal caspase-like domain of separase from *C*. *elegans* in complex with a proposed substrate peptide

To gain information about the degree of structural conservation between subfamilies of CD clan peptidases, we overlaid the crystal structures of human caspase 3 (chains A and B, PDB code 3EDQ [26]), gingipain R (subdomain B, PDB code 1CVR [25]) and human MALT-1 (PDB code 3UO8 [28]). Residues of the catalytic dyad and secondary structure features aligned very well in all three structures (Fig 2A). Gingipain-R contains an additional helix (helix α3’ in Fig 1B) connecting its subdomain A with its subdomain B. This helix is replaced by two extended stretches in caspases called intersubunit linker that enables interaction of their smaller and larger the subunits. This helix is also not formed in human and mouse MALT-1 structures (PDB codes 3UO8, 3V4L) but instead is a region of extended conformation, which was described by Yu and colleagues as an uncleaved intersubunit linker [28]. The active sites of the proteins show very similar conformations despite their different substrate specificities in P1 (Fig 2B). Caspases recognize an acidic aspartate residue in their S1 pocket whereas gingipain-R and MALT-1 recognize the basic residue arginine. When examining the active sites we noticed that in caspase 3, the P1 residue aspartate of the substrate inhibitor ace-LDESD-CHO is locked into position via hydrogen bonds formed to guanidine groups of Arg64 and Arg207 (Fig 2B and S1 File). In gingipain-R, the side chain of the P1 residue arginine is locked into place by forming hydrogen bonds to the side chain of Asp163. In MALT-1, the positively charged P1 residue arginine in the inhibitor VRPR interacts with the side chain of Asp365, Asp462 and Glu500. When superimposing the structures it emerges, that the anchoring residues in caspase 3 (Arg64) and gingipain-R (Asp163) are located in very similar positions in the respective crystal structures, after β-strand β1, at the beginning of helix α1. In MALT-1, Asp365 is located a little further along at helix α1, causing P1-Arg to be drawn further inside the protein than in the case of gingipain-R. The respective P1 residues are anchored by two further sites, one in the loop located between β4 and α5 (R207 for caspase 3, E500 for MALT-1 and main chain oxygen of W284 for gingipain-R) and one at the end of β4 (D462 for MALT-1 and Q161 for caspase 3).

**Fig 3:**
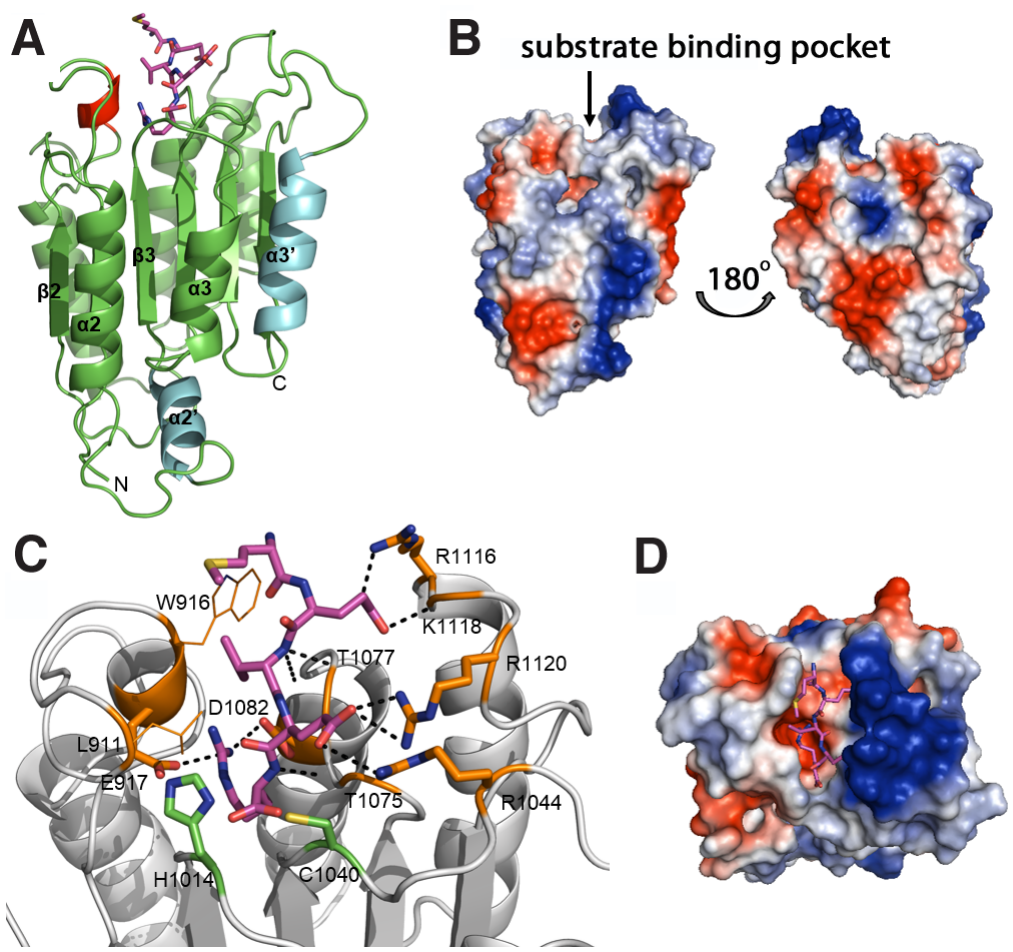
Homology modelling of the caspase-like domain of *C*. *elegans* separase using caspase 3 as template. (A) The homology model of the caspase-like domain of separase from *C*. *elegans* is shown in cartoon view with the proposed substrate peptide MEVER as sticks (magenta). The additional helices α2’ and α3’ that were introduced into the caspase 3 template (cyan) as well as the short helix modelled into the loop between β1 and α1 (red) are highlighted. Core model and loops were generated using MODELLER and energy minimized using GROMACS. (B) Surface electrostatics of the caspase-like domain in separases (shown here without substrate peptide) shows several positive (blue) and negative (red) patches that may be important for interactions with modulators such as securin or other domains within separase itself. Left: front view, same as view in (A). Right: view from back of molecule via vertical rotation by 180°. (C) Proposed interaction of the separase active site with substrate peptide MEVER (in sticks, magenta). Residues interacting with the peptide are labelled and shown in orange. Hydrogen bonds formed between peptide and protein are shown as black dashes. The catalytic residues H1014 and C1040 are indicated as green sticks. (D) Electrostatic properties of the substrate pocket reveal a deep negative pocket that receives the P1-Arg residue and a large positive patch that locks the side chains of P2-Glu and P4-Glu in place. All figures were prepared using PyMol.

For comparative modelling of the catalytic domain of *C*. *elegans* separase we chose caspase 3 (PDB code 3EDQ and [26]) as template because firstly the presence of subdomain A causes a slightly open and twisted conformation of subdomain B in gingipain-R, and secondly, caspases present a more compact fold with shorter loops than human MALT-1. Due to the low sequence identity and similarity between the two proteins, 7.33% and 20.33%, respectively (SIAS server), protein structure analysis was used to generate an accurate alignment. First, the positions of the catalytic residues His and Cys as well as residues predicted to represent the buried β-strands were aligned. Then the positions of other secondary structure elements were aligned and residues thought to interact with the substrate were matched (Fig 3A). Helices α2’ and α3’ were inserted into the template structure file as separate helices and positions adjusted by iterative modelling rounds. The domain boundaries of the caspase-like domain in *C*. *elegans* separase were determined to be Tyr902 and Gln1140. This initial model was enhanced by adding a proposed substrate peptide, which enabled detailed analysis of the substrate pocket and its recognition of cleavage sites. Reports in the literature suggest that the consensus sequence for recognition by separases is D/ExxR with the P1 residue being an arginine, similar to gingipain-R and MALT-1, but in contrast to caspases where the recognition sequence is D/ExxD. In-depth substrate specificity analysis on *S*. *cerevisiae* separase revealed explicit restraints for certain amino acids in positions P2 to P6 and a consensus sequence of SIEVGR [36]. Sullivan and co-workers also suggested that the difference in specificity between budding yeast and human separase is likely due to the absence of a hydrophobic residue in the P5 position in human Scc1-consensus sequence, which is not tolerated by budding yeast separase. Additionally, positions P2 and P3 are occupied by larger hydrophobic residues in human Scc1. This might contribute towards discrimination of these two homologues by separases from budding yeast and human, respectively. The cleavage sites in human Scc1 are DREIMRE and IEEPSRL, in *X*. *laevis* DREMMRE (putative), in *D*. *melanogaster* TPEIIRC (putative) and DREIMRE in mouse (putative) [31]. The recognition sites in *S*. *cerevisiae* are SLEVGRR and SVEQGRR [7]. We aligned Scc1 sequences from different species (S2 File) and determined that the likely recognition sites present in Scc1 in *C*. *elegans* are LMEVERD^200^ or EVERDRD^202^ (Table 1). Interestingly, both proposed cleavage sites in *C*. *elegans* Scc1 possess an acidic residue in position P2, which is not present in other recognition sequences and might be important for the recognition of this sequence by separase from *C*. *elegans*. We therefore included the proposed substrate peptide MEVER in our homology model.

**Table 1:** Putative and confirmed separase substrates and their cleavage sites for different organisms.

Species	Substrate	Recognition sequence	Reference
*H*. *sapiens*	Scc1	DREIM**R**, IEEPS**R**	[31]
*H*. *sapiens*	separase	EWELL**R**, SFEIL**R**, GPEIM**R**	[33, 42]
*M*. *musculus*	Scc1	DREIM**R** (putative)	[31]
*X*. *laevis*	xRad21	DREMM**R** (putative)	[31]
*X*. *laevis*	separase	CEVL**R**	[7]
*D*. *melanogaster*	Rad21	TPEII**R** (putative)	[31]
*S*. *pombe*	Rad21/Scc1	SIEVG**R**, SIEAG**R**	[36] [30]
*S*. *cerevisiae*	Scc1	SLEVG**R**, SVEQG**R**	[7]
*S*. *cerevisiae*	Slk19	SIDYG**R**	[43]
*C*. *elegans*	Scc1	LMEVE**R**, EVERD**R**	

The final model shows a globular protein in which a central six-stranded β-sheet is flanked by seven helices in total, three on each face of the sheet and an additional helix (helix α2’) on the side opposite to the active site (Fig 3A). The core of the protein is supported by hydrophobic interactions, and side chains in neighbouring helices interacting via hydrogen bonds. There are several larger loops present that were modelled iteratively to encourage hydrophobic interactions and hydrogen bonds. One such network of hydrogen bonds links β1, β2 and β3 with the loop between α1 and β2 and the loop between α4 and α5 utilising side chain atoms of Asn959, Tyr961, Tyr904, Arg1006, Gln950 and Lys1097. Another network could be established between side chains of residues Asp1007, Thr903 and Thr1030 thus linking β1 with β3 and the loop leading up to β4. A short helix of one turn was modelled into the large loop between β1 and α1 (α1’). It is supported by hydrophobic interactions of its residues Ile921 and Phe922 with Ile963 at the C-terminal end of β2, Val906 and Cys908 at the C-terminal end of β1 and Ile 1012 and Phe1010 at the C-terminal end of β3. Furthermore, polar interactions are made between main chain amide-NH of Ile921 and carbonyl-oxygens of Arg918 and Glu917 and side chains of Gln913 and Asp923. The model’s dihedral angles were analysed using a Ramachandran plot, in which 88% of amino acids (210) were in preferred regions, 6% (14) in additionally allowed regions and 6% (15) in disallowed regions.

### Substrate specificity in *C*. *elegans* separase

Substrate residues P1 to P5 are thought to be responsible for substrate specificity in caspase and separases [36, 44, 45]. Based on comparison with known co-crystal structures of caspases or caspase-like proteins with peptidic inhibitors and multiple sequence alignment of separase with human caspase 3, gingipain-R and human MALT-1, we hypothesize that separases might make similar interactions with their substrate Scc1 and derived the following main interaction points between protein and peptide substrate.

**Fig 4:**
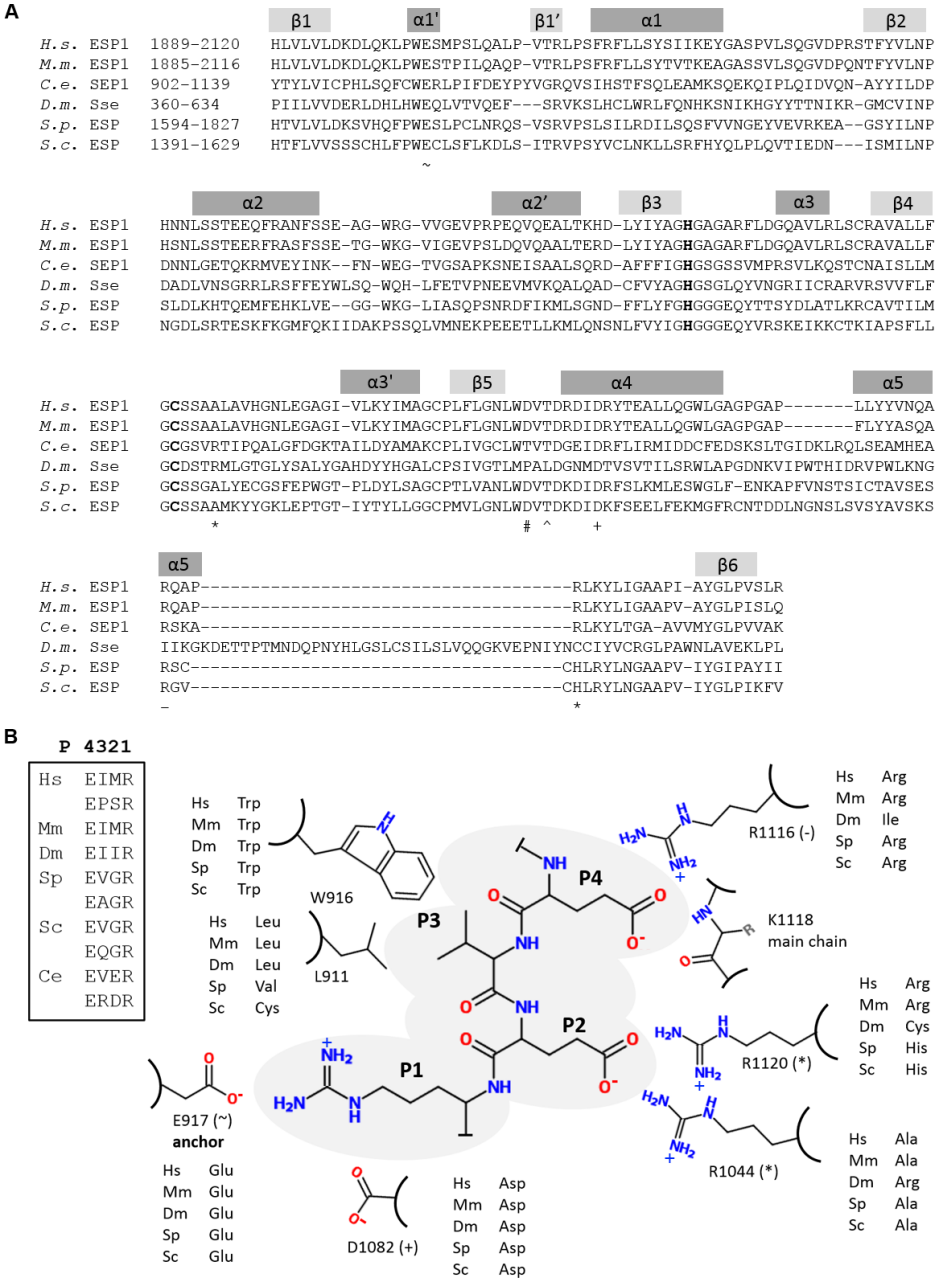
Interactions made between the predicted substrate peptide MEVER and the homology model of the *C*. *elegans* separase caspase domain. (A) Residues in separases that interact with the substrate peptide are highlighted in an alignment. P1-Arg is anchored via hydrogen bonds to Glu917 (~) and Asp1082 towards the N-terminus of helix α4 (+). The main chain amide-NH of residue P1 interacts with main chain oxygen atom of Thr1075 (#). The side chain of P2-Glu forms hydrogen bonds to Arg1120 (which is located in a well-conserved region preceding β6) and Arg1044 (which is located in a loop region between helix α3’ and β4) which are both highlighted with * in the alignment. Aside from hydrophobic interactions, P3-Val interacts with both the main chain and side chain oxygen atoms of Thr1077 (^). P4-Glu forms hydrogen bonds to the main chain amide of Lys1118 and guanidino group of Arg1116 (–). α-helices are shown in dark grey and β-strands in light grey. The catalytic dyad (C, H) is shown in bold letters. (B) Schematic representation of interactions between *C*. *elegans* separase and the proposed substrate peptide EVER. Corresponding interacting residues in separases from other species are taken from Fig 4A. Core recognition sites of Scc1 proteins are shown in the boxed inset.

Gingipain-R and MALT-1 recognize the substrate P1-Arg through side-chain interactions with an aspartate residue (Asp163 and Asp365, respectively) that is located in helix α1 (Fig 2B). In contrast, the conserved acidic residue in this region of separase is a glutamate (Glu917 in *C*. *elegans*, highlighted ~ in Fig 4) located at the beginning of an insertion between β1 and α1 that is predicted to contain a short helix (α1’) and a short β-strand (β1’). We therefore modelled Glu917 into a short helix and positioned the P1-Arg of the predicted substrate peptide, so that the side chains of these two residues interact to closely resemble the situation in substrate complexes of gingipain-R and MALT-1. Another interaction is formed between P1-Arg and the side chain of Asp1082, a conserved residue in helix α4 (highlighted + in Fig 4). This is a similar spatial position to Arg207 for caspase 3, Glu500 for MALT-1 and main chain oxygen of Trp284 for gingipain-R, which form interactions with the P1 side chains of their respective substrates. The base of the substrate pocket is hydrophobic, due to Met1038, Ile1012, Trp916 and Pro920, and interacts with the aliphatic portion of the arginine side chain. We conclude that recognition of the key conserved P1-Arg in separase substrates is through this hydrophobic pocket and the two conserved acidic residues, similar to gingipain-R (Fig 4B). In contrast, the S1 pockets of caspase 3 and MALT-1 harbour a third anchor point that interacts with the P1 residue, which is absent in the separase model. The main chain amide-NH of P1-Arg interacts with main chain oxygen atom of Thr1075 (marked with # in Fig 4), and an equivalent interaction is also observed in the gingipain-R structure (with Gln282), in MALT-1 (with Ala498) and caspase 3 (with Ser205) and other caspase structures with peptidic inhibitors [46–48]. This residue is in a loop region just after β5 in all structures and is a conserved feature of substrate binding that most likely contributes to binding affinity.

The P2 residue is variable between substrate proteins from different organisms (Table 1, Fig 4B). Unusually, the putative *C*.*elegans* sites uniquely have an acidic residue at P2. Our final model indicates that the acidic side chain of the P2 residue may interact with the basic side chains of Arg1120 (which is located in a well-conserved region preceding β6) and Arg1044 (which is located in a loop region between helix α3’ and β4), locking this residue in place (highlighted * in Fig 4). An arginine residue at the Arg1044 position is rare in separase homologues and most have an alanine instead (see S1 File for the full alignment). The second interaction made by P2-Glu with Arg1120 might also contribute to specificity as it is not conserved in *Drosophila*. Therefore, both Arg1044 and Arg1120 may have to be present to allow binding of a peptide with an acidic residue in P2 position. In contrast, the recognition sequence for human Scc1 possesses a large hydrophobic residue in P2 position, a methionine, which might interact with the small hydrophobic residue alanine that is present in human separase where *C*. *elegans* separase harbours an arginine. Indeed, the recognition of the P2 residue is the most distinctive requirement for substrate recognition, and might provide the basis for development of species-specific inhibitors of separases.

The P3 residue is also variable, and we selected valine, as this is a common variant found in *C*. *elegans*, *S*. *pombe* and *S*. *cerevisiae* substrates. The valine side chain forms hydrophobic interactions with Trp916 and Leu911 in our model and a bulkier P3 residue such as isoleucine or methionine, found in human and *Xenopus* Scc1, respectively, might not fit into the pocket. The P3 residue main chain is locked in place through a hydrogen bond between the amide and the side chain oxygen atom of Thr1077 (highlighted ^ in Fig 4). A similar interaction is observed in human MALT-1 with its peptide, where the main chain amide-NH and oxygen atoms of P3-Arg interact with main chain oxygen and amide-NH of Glu500.

The P4 residue in the separase substrate is a glutamate in all proposed and confirmed recognition sequences for separases from other organisms (Table 1). Interestingly, this residue is an aspartate in some caspase recognition sequences (mainly human caspases 2, 3 and 7), and distinguishes caspases preferring an acidic residue in this position from caspases preferring a hydrophobic residue [44]. The acidic side chain of P4 interacts with a main chain amide-NH at the C-terminal end of helix α5 in many caspases, e.g. in caspase 3 (PDB code 3EDQ) it is Phe250, in caspase 7 (PDB code 2QL5) it is Gln576, in caspase 1 from *Spodoptera frugiperda* (PDB code 1M72) it is Asn267. Therefore, we enabled this residue to form a hydrogen bond to the main chain amide-NH of Lys1118 in our model. In our separase model, P4 may also interact with the side chain of Arg1116 (highlighted—in Fig 4), a basic residue that is highly conserved across most organisms and may therefore be important in substrate binding. The fact that P4 is the same residue in all separase recognition sequences analysed suggests a fundamental role in this context. Finally, in our model, the carbonyl-oxygen of P5-Met may interact with Nε of Trp916, locking the N-terminal end of the recognition sequence in place.

Although we primarily examined substrate specificity for Scc1 proteins, our conclusions are also largely true for autocleavage sites and recognition sites in other proteins. It is likely that factors outside the substrate binding site and the mere binding of the recognition sequence add to substrate specificity, as already suggested by Sullivan and co-workers [36]. These could be pockets near the binding site, which enable binding of Scc1 residues outside the recognition sequence or dynamic features of the binding site and surrounding areas. More structural data and systematic mutagenesis studies are required to satisfactorily determine which residues in Scc1 would contribute most to recognition by separases in different species.

Analysis of the surface electrostatics revealed several electropositive and electronegative patches (Fig 3B). Intriguingly, one face of the protein containing helices α2, α2’, α3 and α3’, shows large electropositive patches whereas the other face, helices α1, α4 and α5, harbours electronegative patches. This arrangement suggests that both sides of the protein might provide interfaces for different interaction partners, such as securin or other domains of the separase protein itself. An additional large electronegative patch is located at the base of the parallel β-sheet. It contains acidic residues present in the extended loop between α2 and α2’, which might also be important for binding of an interaction partner. Contrary to the other patches, the additional large electropositive patch that lies near the substrate binding site may mainly be involved in substrate binding. Analysis of the electrostatic properties of the substrate binding site (Fig 3D) shows that the S1 site is a deep electronegative pocket that is well-suited to interact with the P1-Arg residue. The right side of the pocket harbours several electropositive residues Lys1118, Arg 1120 and Arg 1044 that potentially interact with the acidic residues of the substrate, P2 and P4, and lock the peptide in place. The base of the substrate pocket and the left side are largely uncharged owing to the presence of several hydrophobic residues that interact with P3-Val.

### The central region of separases may harbour a death domain fold

**Fig 5:**
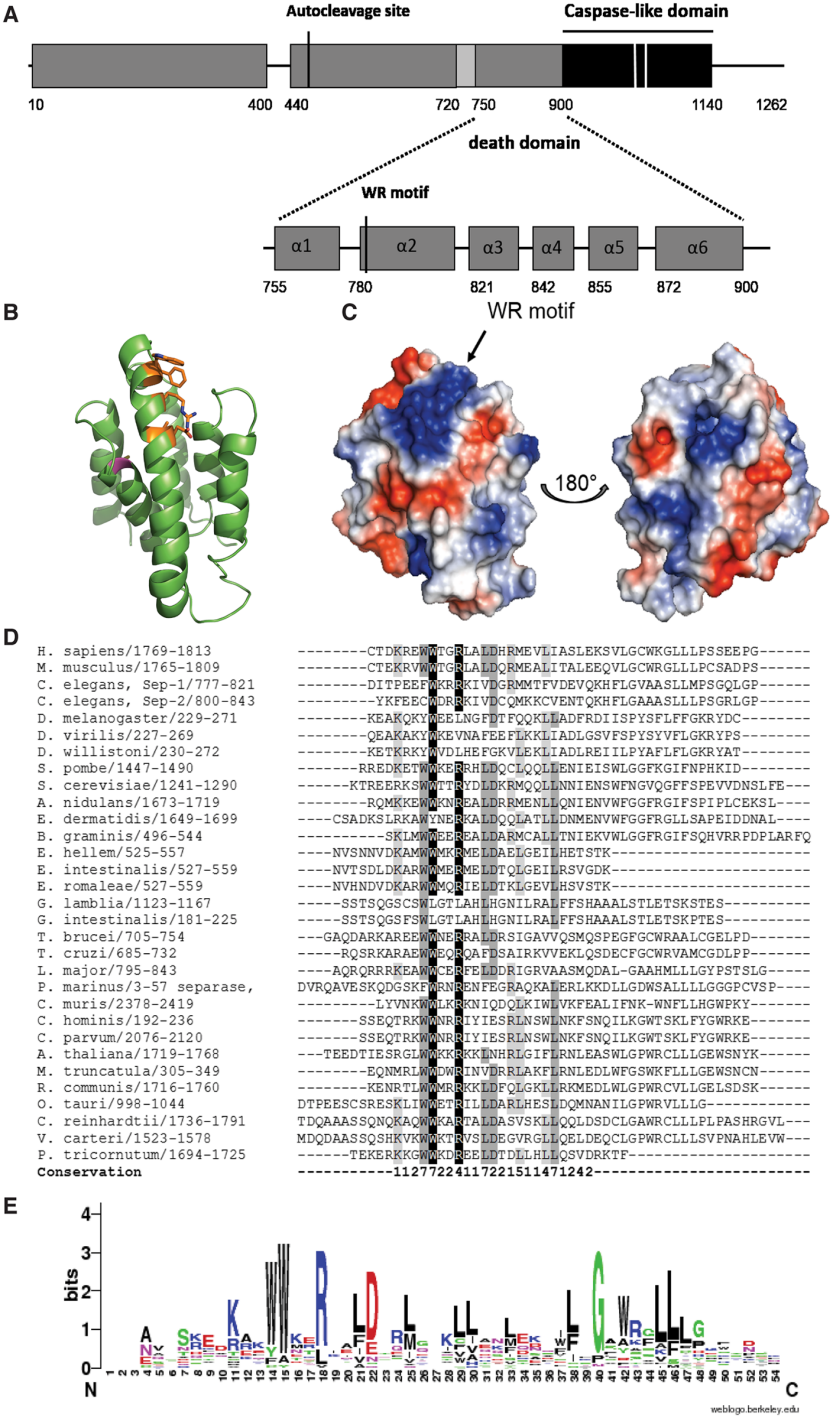
The central region of separases may be similar to death domains and harbours a conserved WWxxRxxLD motif. (A) The region N-terminal to the catalytically active caspase-like domain (black) is made up of six α-helices (grey) and may be structurally similar to death domains. A novel WWxxRxxLD-motif was found in the second helix of this domain whose function remains to be elucidated. Helices are numbered and indicated as grey bars, and their boundaries in *C*. *elegans* separase annotated. The region encompassing three β-strands is shown in light grey. Catalytic residues are marked with white bars. (B) Three-dimensional model of the proposed death domain in separase from *C*. *elegans* using the prodomain of human procaspase-9 as template. The six-helix bundle is shown in cartoon view with amino acids belonging to the proposed the WR motif shown as sticks (orange). A surface-exposed cysteine, C866 is indicated in magenta. Figure prepared with PyMol. (C) Surface representation and electrostatics of the proposed CARD domain show a large electropositive patch where the WR motif is located. Left: front view, same as view in (B), Right: view from back of molecule via vertical rotation by 180°. Figure prepared with PyMol. (D) Sequence alignment of the novel WR motif shows their high conservation within the central region of separase proteins. Sequences from mammals (*Homo sapiens*, *Mus musculus*), *Caenorhabditis elegans*, insects (*Spodoptera frugiperda*, *Drosophila melanogaster*, *Drosophila virilis*, *Drosophila willistoni*), fungi (*Schizosaccharomyces pombe*, *Saccharomyces cerevisiae*, *Exophiala dermatitidis*, *Blumeria graminis*), microsporidia (*Encephalitozoon hellem*, *Encephalitozoon intestinalis*, *Encephalitozoon romaleae*), protozoa (*Giardia lamblia*, *Giardia intestinalis*, *Trypanosoma brucei*, *Trypanosoma cruzi*, *Leishmania major*, *Perkinsus marinus*, *Cryptosporidium muris*, *Cryptosporidium hominis*, *Cryptosporidium parvum*), plants (*Arabidopsis thaliana*, *Medicago truncatula*, *Ricinus communis*) green algae (*Ostreococcus tauri*, *Chlamydomonas reinhardtii*, *Volvox carteri*) and the diatom *Phaeodactylum tricornutum* were aligned. Highlighted residues have 80% or more sequence identity (white letters on black background), 60–80% sequence identity (grey), or 40–60% (light grey). ‘Conservation’ indicates the degree of conservation of physico-chemical properties in each column of the alignment and is represented by numbers from 0 to 10. (E) Weblogo representation of the second predicted helix of the proposed CARD domain. The overall height of the stack indicates the sequence conservation at that position, while the height of symbols within the stack indicates the relative frequency of each amino acid at that position.

Secondary structure prediction on sequences immediately N-terminal of the caspase domain predicted six α-helices, and multiple sequence alignment of the respective regions in different separases reveals conservation of hydrophobic residues at certain positions (S3 Fig). In separase from *C*. *elegans*, helices were predicted from residues 755 to 772 (α1), 780 to 812 (α2), 821 to 835 (α3), 842 to 849 (α4), 855 to 866 (α5) and 872 to 890 (α6) (Fig 5A). Interestingly, this region is also identified as part of the peptidase_C50 family (separases, PF03568, E-value of 2.3e-08) when submitted to the Interpro server [49] indicating that this region is well conserved despite the fact that it is not part of the protease domain. This led us to conclude that separases possess an α -helical domain immediately N-terminal to the caspase-like domain (Fig 5A). This domain may interact with and possibly stabilise the catalytically active protease domain as seen in related proteins. For instance, human MALT-1 is a multi-domain protein that contains both a death domain and two immunoglobulin-like domains (Ig) N-terminal to the caspase-like protease domain (Casp) and a third immunoglobulin-like domain C-terminal. The isolated caspase domain hMALT1_Casp_ (329–566) yielded only oligomeric, poorly folded protein whereas the truncation variant hMALT1_Casp-Ig3_ (334–719) could be expressed in *E*. *coli* and was used to solve the hMALT1_Casp-Ig3_ apostructure [27]. The catalytically active caspase domain in gingipain is preceded by an inactivated domain (subdomain A) that forms a covalently linked dimer with the C-terminal domain, in an arrangement that resembles caspase dimers [25]. Moreover, some of both inflammatory and initiator caspases contain one or more death domains (DD), death effector domain (DED) or caspase recruitment domain (CARD) in their N-terminal regions [39, 50] and oligomerise for activity [51]. These domains are collectively known as death domain superfamily, different subclasses of which have very different sequences but share a common, globular fold made up of six anti-parallel α-helices. Conserved hydrophobic residues at certain positions compose the hydrophobic core of these domains. Considering the importance of death domains for recruitment and activation of caspases and the fact that we predicted six α-helices N-terminal of the caspase-like protease domain led us to investigate the possibility of the presence of a death domain in separases, despite the lack of significant sequence similarity to any known member of the superfamily. As structurally this region might represent a subclass of the death fold domain superfamily, we modelled this region on the CARD domain of human procaspase 9 (PDB code 3YGS) (Fig 5B) to gain further structural and functional insight. Helix α2 was extended in accordance with secondary structure predictions. The model was subsequently adjusted to satisfy positions of amino acids thought to be part of the hydrophobic core: Leu10 (α1), Trp27, Leu30, Leu31 Leu35 and Trp36 (α2), Ile44 (α3), Leu59 and Ile60 (α4), Phe74 and Leu78 (α5), Leu86 and Leu90 (α6) (numbering according to the CARD domain of human procaspase 9). The model shows a six-helix bundle in which the second helix is slightly bent to allow shielding of hydrophobic residues (Fig 5B). Analysis of dihedral angles using a Ramachandran plot revealed that 91% of amino acids (123) were in preferred regions, 5% (7) in additionally allowed regions and 4% (5) in disallowed regions.

Based on the alignment using separase sequences from divergent species we identified a region around residue 783 in *C*. *elegans* separase and 1776 in human separase with the consensus sequence KAKWWKERxALDTRLGKLL (Fig 5D). We termed this region WR motif and determined an overall sequence motif of WWxxRxxLD using Weblogo [52] (Fig 5E). The variable residues x are somewhat different in each species. For example, in *Caenorhabditis elegans* the motif consists of FWKRRKIVD^791^, in *Schizosaccharomyces pombe* this sequence is WWKERRHLD^1462^ whereas in human separase it is WWTGRLALD^1783^. Secondary structure prediction assigns a helix to this region but the significance of this conserved region is yet unknown. However, its high degree of conservation in a previously thought highly divergent part of separase suggests an important role in separase structure or function.

### The N-terminal region of separases consists mostly of α-helices

The N-terminal region of separase is thought to consist of helices, which may be assembled into ARM or HEAT repeats [19, 29] or tetratricopeptide (TPR) repeats [53]. Fold recognition predictions carried out using HHpred [54] and Phyre^2^ [55] matched the N-terminal regions of separases from *H*. *sapiens*, *M*. *truncatula*, *S*. *cerevisiae* and *C*. *elegans* to helical and super-helical structures such as TPR repeats and, with less confidence, to ARM or HEAT repeats. We determined hits to the TPR motif of DNAJ homolog subfamily C member 3 (3IEG_A), E3 SUMO-protein ligase ranbp2 (4GA2_A), TPR repeat of Lipoprotein NLPI (1XNF_A), a synthetic consensus TPR protein (2FO7_A) and a putative peptidylprolyl isomerase (3RKV_A). We could not detect sequence similarities to proteins containing HEAT or ARM repeats with confidence. However, prediction of HEAT or ARM repeats is challenging because of their great sequence variability [56], and we therefore term the N-terminal regions of separases as helical regions, rather than as containing HEAT or ARM repeats. Moreover, the N-terminal regions of separases from different species appear to be of varying lengths, which makes the assignment of a repeat motif difficult. Additionally, some separases contain three β-strands in their N-terminal region and a large unstructured region (Fig 1A). Generally, the N-terminal regions of separases are highly divergent between species, and only future structural studies will determine the individual molecular make-up of these regions and to which class of helical repeats the N-terminal region of separase belongs to.

## Discussion

The C-terminal region of separase contains a catalytic domain belonging to the CD clan of proteases, including caspases, MALT-1 and gingipain-R. Specifically, we propose that the conserved C-terminal domain of separases is very similar to the joint smaller and larger subunits of caspases (p10 and p20, respectively) and gingipain R, subdomain B, and we refer to it as a caspase-like domain. In contrast to Viadiu *et al*. [29] we could not detect a second, inactive caspase-like domain. Our secondary structure predictions do not indicate the presence of a second region of six β-strands (as expected for another heterodimer) or of four β-strands (as expected for an additional larger subunit similar to caspases) N-terminal to the caspase-like region we detected. This discrepancy may be due to the recent availability of separase sequences from a wide range of taxa which allows improved multiple sequence alignments. It is known that initiator caspases function as dimers [51], MALT-1 is more active in an oligomeric state [57] and gingipain contains a subdomain A that is similar to its active subdomain B [25]. In these contexts, self-association may contribute to stabilisation of the protease active site, which leads to an enhanced catalytic activity. However, separases do not possess a second, inactive caspase-like domain which raises the question of whether the single caspase-like domain of separase self-associates to stabilize its catalytic site. Although there is no evidence that separase acts as a dimer, this possibility has not been investigated, and should be borne in mind in future biochemical studies. On the other hand, our analysis also suggests that, similar to many caspases, separases may have a death superfamily domain N-terminal to their catalytic domain. The conservation of these two domains suggests evolutionary pressure to keep them together for separase stability and/ or function. For example, the interaction between the two domains may be facilitated by complementarity of the surface electrostatics of the two domains as was suggested for CARD/CARD interactions [58–60]. Vacuum electrostatic calculations of both the caspase-like domain as well as the death domain showed regions with basic character, particularly around the WR motif (Fig 5C). This might present a binding site for an acidic patch formed by the caspase-like domain of separase or securin, similar to the interactions made between CARD domains in caspases and their respective interaction partners, as described in [61, 62]. Previous studies showed that securin blocks the active site for substrate peptides and contacts residues outside the active site [17, 33]. As an example, interaction experiments both in fission yeast [63] and budding yeast [17] showed that the central and C-terminal part of securin interacts with separase. Horning *et al*. showed that securin efficiently hinders binding of a substrate to the separase active site but binding of securin to separase’s C-terminal region is weak [17]. They therefore suggested an inhibition mechanism where securin distorts the active site. Nagao *et al*. identified a fragment of securin, residues 81 to 156 that physically interacted with separase via a DIE motif [63]. This motif is also present in securins from *S*. *cerevisiae* and *C*. *elegans* and is located in an acidic region within securin. Recently, Han *et al*. identified His134 in human securin to be important for separase binding and initialisation of proteolytic activity [64]. This residue is just C-terminal of the previously identified DIE motif in the acidic region within securin therefore stressing the importance of this region for separase binding and activation. Death superfamily domains are protein-protein interaction modules that activate proteases through the assembly of complexes. The presence of a death domain in separase suggests, therefore, that activation of its catalytic activity may be dependent on self-association. In this scenario, securin could block separase self-association to inhibit activity.

Our model of the caspase-like domain of *C*. *elegans* separase with a proposed substrate peptide (sequence MEVER) is consistent with the predicted recognition sequences for separase from *C*. *elegans* (LMEVER/D^200^ or EVERDR/D^202^). In contrast to other known separase recognition sequences, these harbour an acidic residue in P2 position that is recognised by a pair of arginines. Comparison with known structures of caspases, MALT-1 and gingipain-R bound to peptide inhibitors revealed that while the P1 aspartate residue is locked into position via hydrogen bonding to arginines in caspases, in gingipain-R and MALT-1 this interaction is made by an acidic residue in a similar position. Notably, separases, which also recognize arginine in P1 position, all possess a glutamic or aspartic acid in place where gingipain-R harbours Asp163 in its S1 pocket. Our model suggests that additional interactions between the substrate peptide backbone and protease active site are similar and are therefore likely to be conserved. It is likely that dynamic changes in the substrate pocket also contribute to substrate recognition and specificity, and we believe that this will operate in a broadly similar way to the situation in caspases [65]. To enable analysis of the dynamics of separases that results in solid conclusions will first require the determination of several experimental structures of separase-substrate complexes. This is a priority for our on-going work.

Despite the clear importance of separase in the fundamental processes of chromosome segregation and licensing of centrosome duplication, there has only been a single published structural biology study of this enzyme [29]. We envisage that findings from our study will guide further investigations into the molecular structure and function of this important protein family. In particular, we propose the existence of a death superfamily domain containing a conserved WR motif that lies on the surface of this domain in a position to mediate interactions with other separase domains or binding partners.

## Materials and Methods

### Multiple sequence alignment

Amino acid sequences of separases from different organisms were taken from NCBI. Great care was taken to include as many classes of animals and plants as possible, avoiding a bias of the multiple sequence alignment towards mammals. Separate alignments were carried out for the C-terminal caspase-like domain, the central proposed death domain and the N-terminal domain. Initial alignments were performed in ClustalW [66] using default parameters. Multiple sequence alignments were improved by considering boundaries of known and predicted secondary structure elements and adjusted to increase the alignment of amino acids with similar properties in different sequences using Jalview [41, 67]. We aimed to improve the ‘Quality’ parameter in Jalview which is the BLOSUM62 score based on observed substitutions from 0 to 10 (*). Secondary structure predictions were carried out using both PsiPred [40] and JPred [41].

### Homology modelling

The theoretical models of the caspase-like domain and the proposed death domain from separase of *Caenorhabditis elegans* were generated using Modeller 9.12 [68, 69]. Comparative protein structure modeling in Modeller is done by satisfaction of spatial restraints. The restraints are obtained by assuming that the corresponding distances between aligned residues in the template and the target structures are similar. These homology-derived restraints are usually supplemented by stereochemical restraints on bond lengths, bond angles, dihedral angles, and nonbonded atom-atom contacts that are obtained from a molecular mechanics force field. The model is derived by minimizing the violations of all the restraints using the variable target function method with conjugate gradients, and is then refined using molecular dynamics with simulated annealing as implemented in Modeller [69]. For modelling of the caspase-like domain, human caspase 3 (PDB code 3EDQ) was used as template. The template was adjusted by introducing two helices, α2’ (at the bottom of the caspase-like fold) and α3 (to replace the inter-domain linker). The model of the proposed death domain was calculated using the CARD domain of human procaspase 9 (PDB code 3YGS) as template. Positions of secondary structure elements as well as loops were improved manually and by iterative modelling rounds to satisfy hydrogen-bonding interactions and to shield hydrophobic side chains in protein cores. Both models were evaluated on the basis of geometrical and stereo-chemical constraints using a Ramachandran plot and PROCHECK [70]. Energy minimizations were carried out using GROMACS [71] using steepest descent method with GROMOS96 force field and the SPC water model. The model for the caspase domain was deposited in the Model archive (www.modelarchive.org) as access code ma-ajsa8 and the model for the death domain as access code ma-awsce.

### Analysis of homology models

Surface electrostatics and positions of hydrogen bonds were determined using PyMOL (The PyMOL Molecular Graphics System, Version 1.5.0.4 Schrödinger, LLC.). All figures of structures were prepared using PyMOL.

## Supporting Information

S1 File Multiple sequence alignment of caspase domains. Aligned sequences of caspase domains from caspases, gingipain R and separases from different species.
(TXT)

S2 File Multiple sequence alignment of Scc1 proteins. Aligned sequences of Scc1 proteins from different species. Putative and confirmed cleavage sites are indicated as well as predictions of secondary structure elements.
(TXT)

S3 File Multiple sequence alignment of proposed death domain. Aligned sequences of separases from different species and sequences of known death domains (PDB code indicated). Predictions of secondary structure elements are indicated.
(TXT)
